# Psychological interventions to pregnancy-related complications in patients with post-traumatic stress disorder: a scoping review

**DOI:** 10.1186/s12888-024-05926-6

**Published:** 2024-06-27

**Authors:** Zhuo Peng, Jin Liu, Bangshan Liu, Jiansong Zhou, Li Zhang, Yan Zhang

**Affiliations:** Department of Psychiatry, National Clinical Research Center for Mental Disorders, and National Center for Mental Disorders, The Second Xiangya Hospital of Central South University, Changsha, Hunan 410011 China

**Keywords:** Post-traumatic stress disorder, Pregnant women, Psychotherapy, Complications

## Abstract

**Objective:**

This scoping review sought to investigate the association between pregnancy-related complications and post-traumatic stress disorder (PTSD) among postpartum women, then summarize effective psychological interventions for pregnancy-related PTSD or sub-PTSD.

**Method:**

Publications in English and Chinese were searched in PubMed, Embase, Cochrane, ISI Web of Science, China National Knowledge Infrastructure (CNKI), and WanFang databases using the subject headings of “Stress Disorders, Post-Traumatic”, “Pregnant Women”, and “psychotherapy”. To ensure that as many relevant studies are incorporated as possible, free terms such as prenatal, postnatal, perinatal and gestation were also used. Intervention studies and related cases published by July 1st, 2023, were also searched.

**Results:**

Twenty-one articles (including 3,901 mothers) were included in this review. Evidence showed that typical psychological interventions exhibited great effect, and family support programs, peer support, online yoga, and music therapy were also effective in reducing risk and improving the psychological well-being of the studied population.

**Conclusion:**

Fetal abnormalities, miscarriage, premature birth, infants with low birth weights, hypertension, pre-eclampsia, HELLP syndrome, and hyperemesis gravidarum are associated with an increased risk of PTSD. Moreover, high-risk pregnant women may benefit from psychological interventions such as cognitive behavioral therapy (CBT). It may also be feasible and well-accepted for music therapy and exposure therapy to lessen the intensity of PTSD in mothers.

**Supplementary Information:**

The online version contains supplementary material available at 10.1186/s12888-024-05926-6.

## Background

According to the Diagnostic and Statistical Manual of Mental Disorders, 5th edition (DSM-5), post-traumatic stress disorder (PTSD) is a psychiatric disorder that may occur in people who have experienced or witnessed a traumatic event, series of events or set of circumstances. It is commonly known that there are differences between genders in PTSD, with women suffering at a 2:1 ratio[1]. According to previous studies, pregnancy is recognized as a special period of immunological and physiological change [2], and it is also a period when the chance of PTSD increases and reaches a peak right before labor [3]. Approximately one-third of women in the general population have experienced a traumatic birth, and 3–6% of all postpartum women are diagnosed with PTSD. Furthermore, the prevalence of PTSD in high-risk groups is as high as 18.9%, but many of them remain undiagnosed [4, 5]. PTSD symptoms can affect mother–child interaction and may be responsible for the mothers’ social isolation [6, 7].

Pregnancy-related complications include physical and mental conditions that affect the health of pregnant or postpartum women and/or their babies. Physical and mental conditions that can lead to complications may start before, during, or after pregnancy [8]. Common complications include gestational hypertension, diabetes, nausea, vomiting and anemia, and serious complications include miscarriage, premature birth, premature rupture of membranes, stillbirth, low birth weight, birth defects, and infant and maternal morbidity or death [8]. Severe pregnancy-related complications such as pre-eclampsia (PE), HELLP syndrome, and hyperemesis gravidarum (HG) may increase the risk of PTSD and physical consequences. In the first trimester, pregnant women who experience miscarriage, stillbirth, and fetal abnormalities are found more likely to have emotional consequences such as depression, anxiety, and distress, which, however, are often overlooked [9]. In the second and third trimesters, pregnant women may face preterm birth, premature rupture of membranes, babies with very low birth weights, and others. Pregnancy-related complications have been found associated with PTSD as well as other injuries. In addition to raising the incidence of maternal PTSD, PE may also be linked to brain damage that impairs memory, and women with PE may frequently experience cognitive dysfunction [10, 11]. Nausea and vomiting during pregnancy can be persistent in 2% of pregnant women and worsen to develop into HG [12]. Post-traumatic stress (PTS) symptoms are common after HG [13]and may have long-term implications on a mother’s mental health. Symptoms of depression, anxiety, and PTSD may still be present through years after HG [14]. Among women who had experienced miscarriage, 28% may develop PTSD at 1 month after miscarriage, 0.6–5% may develop PTSD at 3 months after miscarriage, and 18% may develop PTSD at 9 months after miscarriage [15]. It was also found that approximately 50% of women who experienced abortion became pregnant again within 12 months after their abortion [16]; however, a prior abortion is also found to be a risk factor for anxiety, depression and PTS during subsequent pregnancies within one year [16]. Moreover, prior abortions have also been found associated with poor neuropsychiatric outcomes in offspring [17–19].

Early psychological intervention may help buffer parental influence on child outcomes [20], and it also plays a crucial role in reducing postpartum morbidity [21]. Early psychological intervention involves psychological, physical, and drug therapies. However, with regard to pharmacotherapy, doctors may hesitate to prescribe psychotropic medications due to potential side effects on mothers and their babies. There is also evidence that pregnant women prefer psychotherapy to pharmacotherapy or other types of psychiatric intervention [22]. Recurrent exposure to trauma may further sensitize underlying and latent illnesses, therefore, early psychological intervention may help avoid the buildup of unprocessed traumatic memories [23]. The National Institute for Health and Care Excellence (NICE) recommends that trauma-focused cognitive behavioral therapy (CBT) and extended exposure treatments be initiated within four weeks after the occurrence of traumatic events. For patients whose traumatic experience happened four weeks prior, eye movement desensitization and reprocessing (EMDR) is also a good option [24]. Previous studies have suggested that a variety of psychological interventions might be effective in perinatal pregnant women with PTSD, however, no conclusion has been reached regarding the effectiveness of different psychological interventions on this special population. Therefore, we conducted this scoping review to summarize the effective psychological interventions for patients with maternal PTSD resulting from pregnancy-related complications.

## Methods

### Research question/objective

Question 1. What are the main pregnancy-related complications that may precipitate PTSD or sub-clinical PTSD in affected mothers?

Question 2. What psychological interventions can be implemented for mothers experiencing PTSD or sub-clinical PTSD resulting from pregnancy-related complications?

Question 3. What is the efficacy of these psychological interventions in mitigating PTSD or sub-clinical PTSD among mothers affected by pregnancy-related complications?

### Search strategy

Publications were searched in PubMed, Embase, Cochrane, Web of Science, CNKI, and WanFang databases, and only those published in English and Chinese were included in this study. See Supplementary Table 1 for details. We listed the keywords based on the topic of the study before literature search and then consulted a panel of professionals to finalize the keywords used in this scoping review. The following medical subject headings and terms (MESH) were used: “stress disorders, post-traumatic”, “pregnant women”, “psychotherapy”, “psychotherap*”, and “behavior therapy”; the terms “maternity,” “prenatal,” “postpartum,” and “perinatal” were also used. All the articles that had been retrieved were then screened according to the inclusion and exclusion criteria.

### Selection of studies

#### Selection criteria

**Fig. 1:**
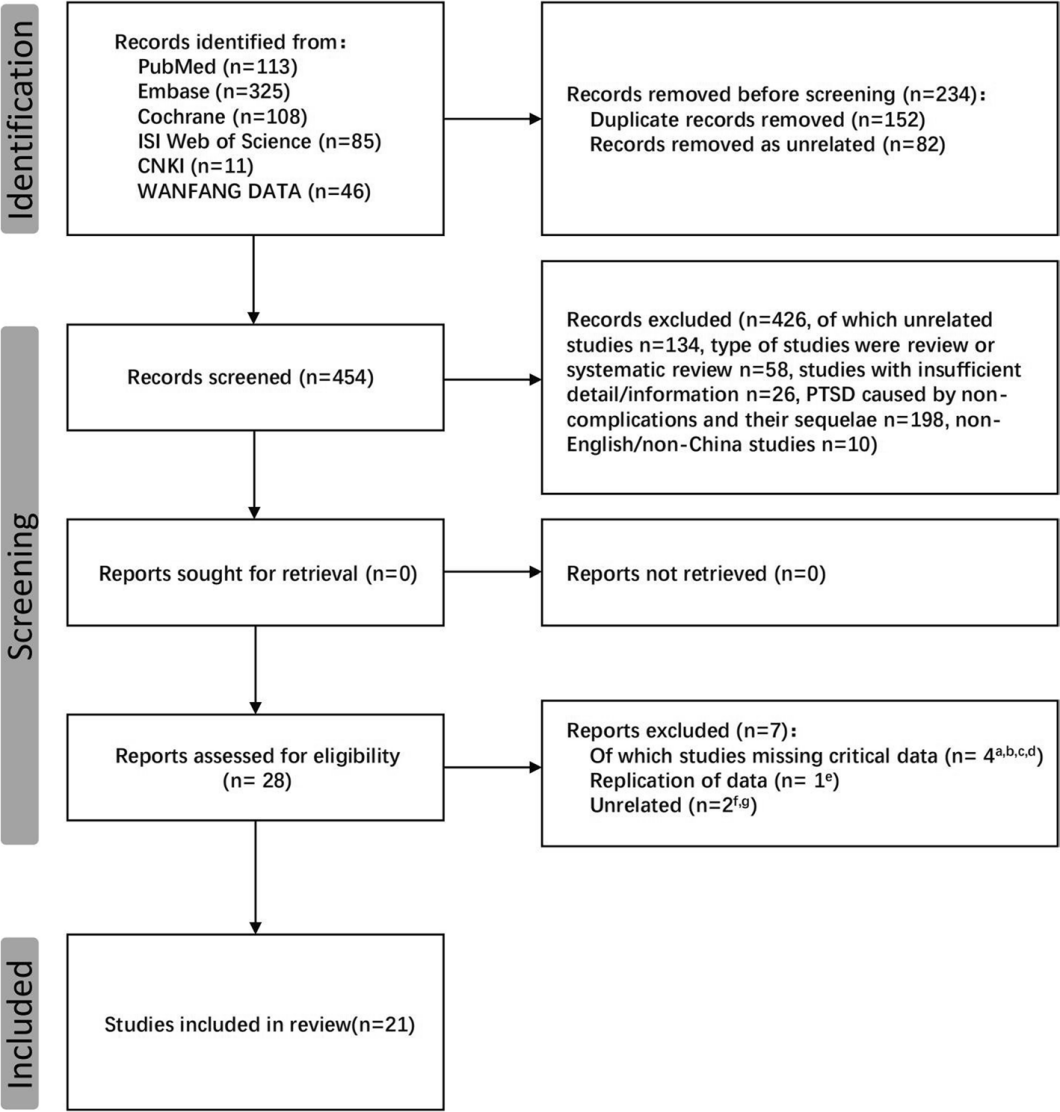
PRISMA flow diagram of the study selection process. a. Nancy et al.; b. Johnson et al.; c. SIMON; d. Zelkowitz et al.; e. Huberty et al.; f. Sjömark et al.; g. Xiuchun Wang et al.

Studies that met the following criteria were included: (1) randomized controlled trials (RCTs), before-after (B-A) studies, prospective cohort studies, cross-sectional studies, and case reports published between January 1, 1996 and July 1, 2023; (2) studies involving women with PTSD caused by pregnancy-related complications; (3) received one or more psychological interventions to prevent and/or treat PTSD resulting from pregnancy-related complications; (4) PTSD or sub-PTSD is diagnosed by using a confirmed and standardized self-report or diagnostic interview as regulated by the Diagnostic and Statistical Manual of Mental Disorders (DSM-IV; 1995; or DSM-V, 2013, APA 2018) or the International Classification of Diseases (ICD-9, ICD-10 or ICD-11, WHO 1993, 2022); (5) publications in English or Chinese. Studies with insufficient information, articles published in other languages, case reports, conference abstracts, and studies on PTSD due to non-pregnancy related complications were excluded. The process of study selection is presented in a PRISMA flow diagram (Fig. 1) [25].

#### Screening process

The screening process consisted of three steps. First, studies were imported into the Rayyan web application (https://www.rayyan.ai/) for duplicate removal and screening. Second, following the inclusion and exclusion criteria, two reviewers (ZP and LZ) in the evidence review group screened the retrieved articles independently based on titles, abstracts, and then full texts. A third reviewer (JL) would be involved to resolve any disagreement through discussion or consultation. A total of 688 records were identified from the six databases. One hundred and fifty-two publications were removed as duplicate records, and 82 were removed due to irrelevance. Four hundred and twenty-six studies were excluded for not meeting the requirements (*n* = 134 for irrelevance, *n* = 58 for reviews and systematic reviews, *n* = 26 for incomplete information and conference articles, *n* = 198 for studies involving PTSD for non-pregnancy reasons, and *n* = 10 for articles published in other languages). We further evaluated the remaining 28 publications: 7 articles were excluded for not meeting the inclusion criteria (*n* = 1 for data duplication and *n* = 5 for missing key indicators) and one article was excluded for irrelevance. Finally, 21 publications were included in this scoping review.

#### Data collection

Data were extracted by two reviewers (ZP and LZ) using the established data extraction forms. The following data were collected: (a) authorship and publication year; (b) study design; (c) country; (d) number of participants; (e) pregnancy-related complications; (f) the questionnaire and timing of PTSD assessments; (g) type and time of interventions, and (h) severity of PTSD (Table 1). A meta-analysis of the included studies was not practical due to the heterogeneity of the patients and procedures (therapies, PTSD questionnaires, and timing of interventions and assessments).

**Table 1:** Description of scoping reviews of psychological interventions for pregnancy-related complications in patients with post-traumatic stress disorder

Authors Year	Type of study	Country	Population	Complications of pregnancy or their sequelae	PTSD assessment	Intervention	Timing of the intervention	Timing of PTSD assessment	Severity of PTSD		Outcomes
									Before intervention	After intervention	
LiMing Wang 2022 [1]	Cross-sectional study	China	Mothers *n* = 115	HDP	PCL-C	-	-	T_0_: baseline (1–24 weeks after birth)	31 .04 ± 6.85	-	13.04% (15/115) were positive for PTSS
YingWu 2023 [2]	B-A	China	Mothers *n *= 90	PE	PCL-C	Empowering education with family involvement	After admission, 60 min/session, twice a week	T_0_: baseline (on admission) T_1_:1d after termination of pregnancy T_2_:1d after discharge	T_0_:50.56 ± 10.37 T_1_:67.98 ± 14.56	T_2_: 51.96 ± 11.53	Significant reductions in avoidance, intrusion, and hyperarousal symptoms (*P *< 0.05)
Claire A.I. Stramrood 2012 [3]	Case	Netherlands	The patient was a 25-year-old gravid 1 para 0	First birth: Pre-eclampsia Second birth: Postpartum Hemorrhage	Clinical Interview	EMDR	The intervention was started after admission and lasted 4 times	-	-	-	No physical symptoms were further reported
YVONNE H. M. POEL 2009 [4]	Case series	Netherlands	Mothers *n *= 24	PE, E and/or HELLP syndrome	Semi-structured interview	No treatment (*n *= 3) Various psychotherapies (*n *= 21)	-	-	Hyperarousal (*n *= 15) Anxiety and feelings of tension (*n *= 10) Re-experiencing problems/symptoms (*n *= 9)	-	20.83% (5/24) were diagnosed with PTSD before intervention Ten (48%) mothers reported to return to their work
Helena Kames Kjeldgaard 2019 [5]	Prospective cohort study	Norway	Mothers n = 1945, G_1_: no nausea (*n *= 574), G_2_: mild nausea(n = 813), G_3_: severe nausea (*n *= 522), G_4_: HG (hospitalised due to nausea, *n *= 20)	HG	P-SS	-	-	T_0_: at 8 weeks after birth T_1_: at 2 years after birth	8w G_1_: 19.8 (18.0; 21.6) G_2_: 20.4 (18.6; 22.1) G_3_: 20.6 (18.8; 22.4) G_4_: 22.6 (19.8; 25.5) 2y G_1_: 18.5 (16.4; 20.7) G_2_: 19.1 (17.0; 21.1) G_3_: 19.3 (17.2; 21.3) G_4_: 21.3 (17.8; 24.8)	-	72 (3.7%) and 35 (1.8%) women reported birth-related PTSD at T_0_ and T_1_, respectively
JieQiong Zhang 2018 [6]	RCT	China	Mothers *n *= 97, Intervention group, *n *= 47 Control group, *n *= 50	Fetal abnormalities, induced labor, stillbirth	IES-R	Internet-based peer support	The intervention was started 1 day after admission and lasted 7 weeks	T_0_: baseline (within 24 h of admission) T_1_: at 42 days after birth	G_1_: 30.85 ± 13.03 G_2_: 29.91 ± 15.16	G_1_: 16.66 ± 7.97 G_2_: 22.69 ± 13.62	Significant reductions in avoidance, intrusion, and hyperarousal symptoms (*P *< 0.001)
RuanZhen Dong 2018 [7]	B-A	China	Mothers *n *= 344, T_1_: Mothers *n *= 344 T_2,3_: Mothers n = 296	Induced labor after adverse pregnancy	IES	Comprehensive psychological intervention:1) reconstruction of cognition; 2) To understand the situation, to improve; 3) Carrying out kind-like nursing	The intervention was started after T_2_	T_1_: within hours of induction of labor T_2_: at 4w after labor T_3_: at 7w after labor	97 (33%) mothers were found to have PTSD	-	Significant reductions in post-traumatic dissociative and peritraumatic emotions (*P *< 0.05) Measure: PDEQ and PEL
Anette Kersting 2011 [8]	RCT	Germany	Mothers *n *= 59, T_1_: Mothers *n *= 26 T_2,3_: Mothers *n *= 33	Abortion, termination of pregnancy due to fetal abnormality, or stillbirth	IES	iCBT	The intervention was started 1 day after admission and lasted 5 weeks	T_0_: before intervention T_1_: after intervention T_2_: 3-month follow-up	G_1_: 33.1(13.21) G_2_: 34.6(11.39)	G_1_: 17.9(12.36) G_2_: 27.9(10.92)	Significant reduction in the severity of PTSS (*P* < 0.05)
Anette Kersting 2013 [9]	RCT	Germany	Mothers *n *= 228, Intervention group, *n *= 113 Control group, *n *= 115	Abortion, termination of pregnancy due to fetal abnormality, or stillbirth	IES-R	Brief Internet-based intervention: structured writing assignments based on cognitive behavioral therapy and the written disclosure procedure	The intervention was started 1 day after admission and lasted 5 weeks	T_0_: before intervention T_1_: after intervention T_2_: 12-month follow-up	G_1_:30.50(12.02) G_2_:31.65(11.59)	G_1_:17.64(12.22) G_2_:28.27(11.81)	Significant reductions in avoidance (*P* < 0.05), intrusion (*P* < 0.001), and hyperarousal (P < 0.05)
Jennifer Huberty 2018 [10]	Cross-sectional study	USA	Mothers *n *= 74, _*_Mothers had a history of stillbirth occurring within the previous 24 months	Stillbirth	IES-R	Online Yoga	The intervention was started 1 day after admission and lasted 12 weeks	T_0_: before intervention T_1_: after intervention	35.43(14.73)	-	38 (51.40%) mothers reported PTS
Ali Navidian 2017 [11]	RCT	Iran	Mothers *n *= 47, fathers *n *= 53 Intervention group, *n *= 50 Control group, *n *= 50	Stillbirth	PPQ	4-session of psychological counseling	The intervention was started 1 day after admission and lasted 2 weeks	T_0_: baseline T_1_: within 4 weeks post-intervention	G_1_:7.22 (4.19) G_2_:7.64 (5.20)	G_1_:4.52 (2.14) G_2_:6.50 (3.28)	Significant reduction in the severity of PTSS (*P *< 0.001)
Shiwen Sun 2017 [12]	RCT	China	Mothers *n *= 80, Intervention group, *n *= 39 Control group, *n* = 41	Fetal malformation and stillbirth	IES-R	Family-support program	The intervention was started 1 day after admission and lasted 6 weeks	T_0_: baseline (within 24 h of admission) T_1_: at 42 days after birth	G_1_:24.46 (13.69) G_2_:23.85 (12.51)	G_1_:13.03 (7.71) G_2_:18.98 (14.62)	Significant reductions in avoidance (*P* < 0.05) and hyperarousal (*P* < 0.05)
ShiWen Sun 2018 [13]	RCT	China	Mothers *n *= 138, Intervention group, *n *= 69 Control group, *n *= 69	Fetal abnormalities	IES-R	Family-support program	1st stage: after admission 2nd stage: after induction of Labor 3rd stage: telephone follow-up performed at 15 days postpartum	T_0_: baseline (within 24 h of admission) T_1_: within 15 days post-intervention	G_1_:24.09土12.88 G_2_:23.81士12.22	G_1_:13.52 ± 7.97 G_2_:19.06 + 15.03	Significant reduction in hyperarousal (*P *< 0.05)
JingJing Wang 2020 [14]	RCT	China	Mothers *n *= 98, Intervention group, *n *= 49 Control group, *n *= 49	Fetal abnormalities: stillbirth, malformation, missed abortion	CAPS	Empathy combined family-support programme	at a time that was convenient to families	T_0_: baseline (within 24 h of admission) T_1_: within 15 days post-intervention	G_1_:105.84 ± 15.43 G_2_:104.13 ± 15.57	G_1_:65.91 ± 6.14 G_2_:78.56 ± 10.48	Significant reduction in the severity of PTSS (*P *< 0.05)
Ayala Borghini 2014 [15]	RCT	Switzerland	Mothers *n *= 55, Intervention group, *n *= 26 Control group, *n *= 29	Preterm birth	PPQ	Three-stage intervention: 1) Joint observation; 2) Videotaped NBAS and semi-structured interview based on the Clinical Interview for Parents of High-Risk Infants; 3) Videotaped mother-infant free play with subsequent IG phase	1st stage: 33 weeks after conception 2nd stage: 42 weeks after conception 3rd stage: 4 months’ CA	T_0_: 42 weeks after conception (> 9 weeks after birth) T_1_: 4 months’ CA T_2_: 12 months’ CA	-	T_0_: G_1_: 4.62(3.54) G_2_: 3.55 (2.88) T_1_: G_1_: 3.31(3.38) G_2_: 3.59 (3.31) T_2_: G_1_: 2.69(2.58) G_2_: 3.17 (2.66)	Mothers of preterm infants showed significantly more PTS (*P *= 0.001) Significant reduction in the severity of PTSS at T_1_ (*P *< 0.05) and T_2_ (*P *= 0.001)
Richard J. Shaw 2013 [16]	RCT	USA	Mothers *n* = 105, Intervention group, *n* = 62 Control group, *n* = 43	Preterm birth and newborn admission to the NICU	DTS	TF-CBT 6-session Intervention Manual (intervention group) One 45-min information session (control group)	The intervention was started 1 day after admission and lasted 3–4 weeks	T_0_: baseline (1–2 weeks after birth) T_1_: within 1-week post-intervention	G_1_: 49.40 ± 25.49 G_2_: 42.35 ± 27.05	G_1_ changed: -12.886 G_2_ changed: –5.509	Significant reduction in the severity of PTSS (*P* < 0.05)
Richard J. Shaw 2023 [17]	B-A (different cases)	USA	Mothers *n* = 26, _*_Mothers of preterm infants (25–34 weeks' gestational age; > 600 g)	Preterm birth	DTS	Group TF-CBT	The intervention was started after baseline and lasted 3–6 weeks	T_0_: baseline (1–2 weeks after birth) T_1_: after 6 weeks post-intervention T_2_: after 6 months post-intervention	48.19 (27.02)	T_0-1_ change: -5.1 T_1-2_ change: -16.1 T_0-2_ change: -21.2	Non-significant reduction in the severity of PTSS (*P* = 0.252)
Susann Kobus 2023 [18]	RCT	Germany	Mothers *n* = 33, Intervention group, *n *= 18 Control group, *n *= 15	Preterm birth	IES-R	Music Therapy	The intervention was started 2 weeks after childbirth and lasted twice a week before discharge	T_0_: baseline (a week after birth) T_1_: after discharge	IES-R Intrusion G_1_:20.3 (16.1–24.6) G_2_:19.7 (15.9–23.6) IES-R Avoidance G_1_: 11.6 (7.1–16.1) G_2_: 13.6 (8.1–19.1) IES-R Hyperarousal G_1_: 18.4 (13.6–23.2) G_2_: 21.1 (15.5–26.8) Total Score: G_1_: -1.2(-2.1-(-0.3)) G_2_: -0.6 (-1.7–0.5)	IES-R Intrusion G_1_: 10.8 (7.1–14.5) G_2_: 16.1 (12.3–19.9) IES-R Avoidance G_1_: 6.7 (3.2–10.2) G_2_: 10.5 (6.8–14.1) IES-R Hyperarousal G_1_: 8.2 (5.4–10.9) G_2_: 22.7 (16.3–29.1) Total Score G_1_: -2.9(− 3.5-(− 2.3)) G_2_: -0.5 (− 1.6–0.6)	Significant reduction in hyperarousal, moderate reduction in intrusion, and mild reduction in avoidance
Stephanie Simon 2021 [19]	B-A	USA	Mothers *n *= 19 (*n *= 13 at six-week follow-up, *n *= 7 at six-month follow-up) Control group, *n *= 0	Preterm birth	DTS	Group TF-CBT	The intervention lasted 3 weeks	T_0_: baseline (1–2 weeks after birth) T_1_: after 6 weeks post-intervention T_2_: after 6 months post-intervention	T_0_: 47.32 (30.41)	T_1_: 41.92 (26.67) T_2_: 24.00 (16.33)	Significant reduction in the severity of PTSS at T_1_ (*P* < 0.05) and T_2_ (*P* < 0.05)
Antje Horsch 2015 [20]	RCT	Switzerland	Mothers *n *= 65, Intervention group, *n *= 33 Control group, *n *= 32	Preterm birth	PPQ	Expressive writing	At 3 months’ CA	T_0_: baseline (3 months’ CA) T_1_: post-intervention (4 months’ CA) T_2_: post-intervention (6 months’ CA)	G_1_:4.09 (2.9) G_2_: 4.14 (3.2)	T_1_: G_1_: 2.60 (2.5) G_2_: 3.64 (3.1) T_2_: G_1_: 2.78 (2.7) G_2_: 4.05 (3.3)	Significant reduction in the severity of PTSS (*P *< 0.05)
Massumeh Koochaki 2018 [21]	RCT	Iran	Mothers *n *= 81, Intervention group, *n *= 42 Control group, *n *= 39 _*_Mothers with a preterm (born before 37 weeks of gestation)	Preterm birth and/or low-birth-weight infant (< 2500 g) hospitalized at the NICU	P-SS	CBT	The intervention was started ≥ 1 month after NICU hospitalization and lasted 4 weeks	T_0_: baseline (≥ 1 month after NICU hospitalization) T_1_: immediately after the intervention (4 weeks after T_0_) T_2_: 3 weeks after the intervention	G_1_:8.095 ± 5.202 G_2_:6.820 ± 4.297	G_1_: 4.547 ± 2.297 G_2_: 6.564 ± 4.290	Significant reduction in the severity of PTSS (*P *< 0.001)

*B-A* before-after study, *RCT* randomized controlled trial, *CA* corrected age, *GA* gestational age, *CBT* cognitive-behavioral therapy, *TF-CBT* Trauma Focus-Cognitive Behavioral Therapy, *iCBT* Internet-based cognitive behavioral therapy, *EMDR* eye movement desensitization reprocessing, *PCL-C* PTSD Checklist-Civilian Version, *PCL-5* PTSD Checklist for DSM-5, *CAPS* Clinician-Administered PTSD Scale, *DTS* Davidson Trauma Scale, *IES* Impact of Event Scale, *IES-R* Impact of Event Scale-Revised, *PDEQ* Peritraumatic Dissociative Experience Questionnaire, *PEL* Peritraumatic Emotions List, *P-SS* PTSD Symptom Scale, *PPQ* Perinatal PTSD Questionnaire, *CAPS* Clinician-Administered PTSD Scale, *NICU* neonatal intensive care unit, *PTSD* post-traumatic stress disorder, *PTSS* posttraumatic stress symptoms, *PTS* posttraumatic stress, *HDP* hypertensive disorders of pregnancy, *PE* pre-eclampsia, *E* eclampsia, *HELLP* hemolysis, elevated liver enzymes and low platelets count syndrome, *HG* hyperemesis gravidarum, *PPROM* preterm premature rupture of membrane

## Assessments of risk of bias

**Fig. 2:**
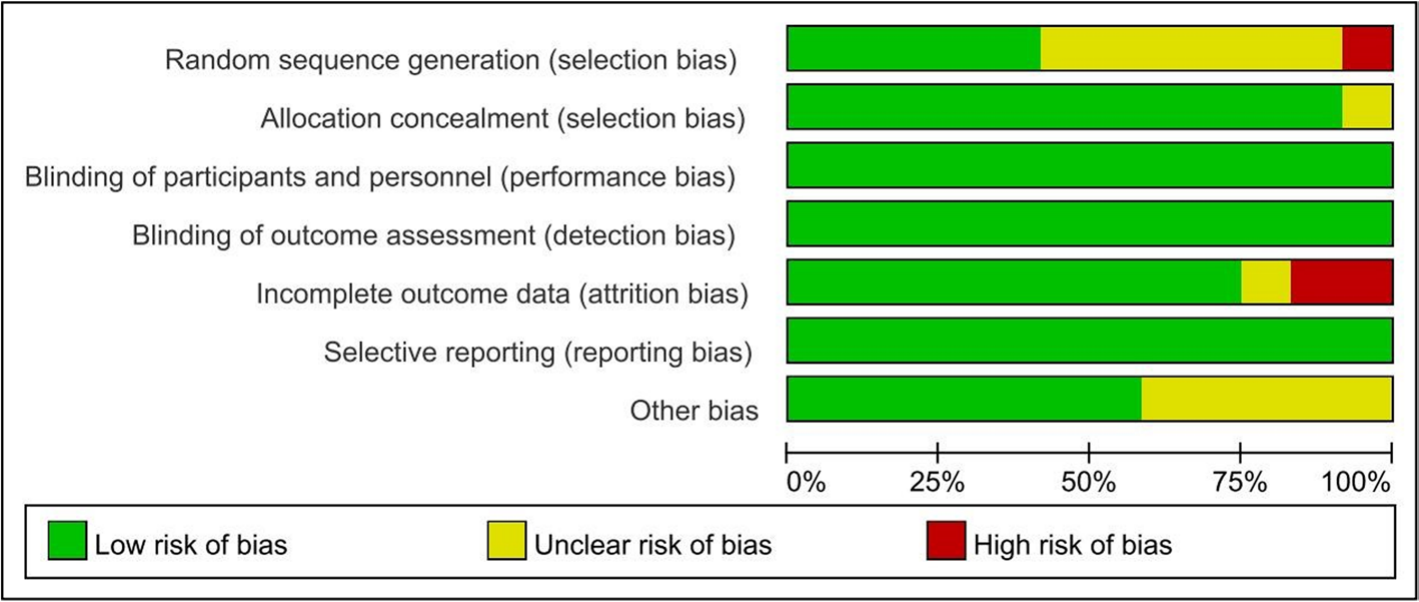
Risk of Bias summary table

**Fig. 3:**
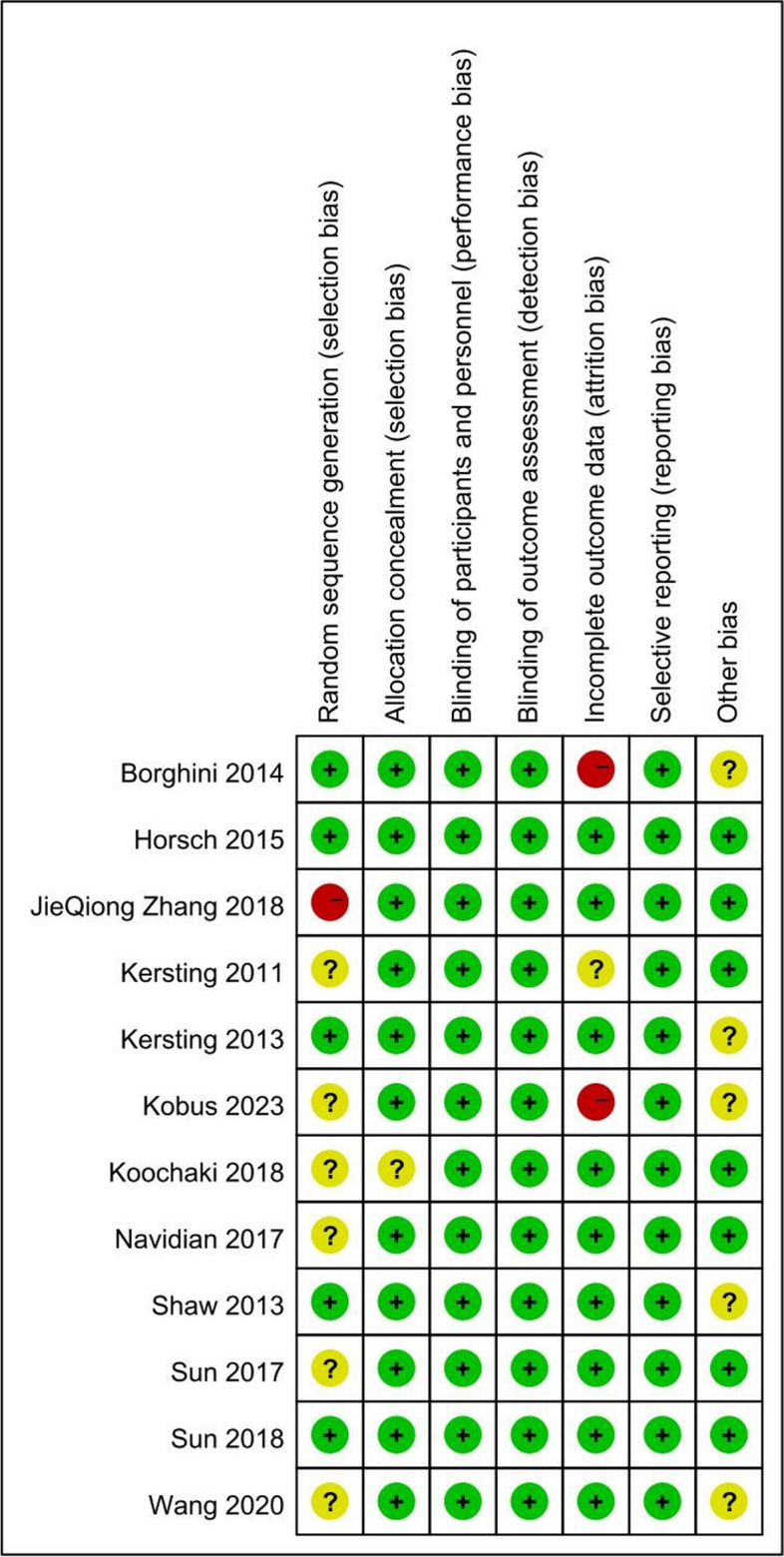
Risk of Bias summary table

The criteria listed in the Cochrane Handbook for Systematic Reviews of Interventions were used to determine the risk of bias. For all the studies included, biases were rated as high, unsure, or low through discussion with the review panel (Figs. 2 and 3). The possibility of biased judgments was considered when evaluating the overall effectiveness of the treatments.

The criteria from the National Institutes of Health (NIH) Quality Assessment Tools for before-after (pre-post), observational cohort, cross-sectional, and case series studies with no control groups (available online at https://www.nhlbi.nih.gov/health-topics/study-quality-assessment-tools) were used to evaluate the quality of the included studies. It contains 9–14 items with response options of “yes”, “no”, “cannot determine”, “not applicable”, and “not reported”, with the objective of directing reviewers’ attention to the essential ideas for assessing the study’s internal validity. Responses to the signaling questions and the relevance assigned to the major ideas represented in the signaling questions were used to determine the quality of the studies (available online at https://www.nhlbi.nih.gov/health-topics/study-quality-assessment-tools).

## Results

### Overview of included studies

A total of 21 studies were included. There are ten RCT studies, three before-after studies, two cross-sectional studies, two prospective studies, three clinical studies, and one case report. Pregnancy-related complications included gestational hypertension (k = 1), pre-eclampsia and HELLP syndrome (k = 3), hyperemesis gravidarum (k = 1), fetal abnormalities (k = 10), and premature birth (k = 7). The conclusions of the studies are shown in Table 1.

### Intervention

## Cognitive Behavioral Therapy (CBT)

CBT is a first-line treatment for PTSD in adults and children. Among interventions for baby loss, short-term Internet-based CBT was found effective [26]. Furthermore, CBT can bring both short- and long-term influence. According to Koochaki et al., CBT has an outstanding effect on preterm mothers immediately and at 3 weeks after intervention, as compared to the control group.

### Trauma-focused CBT (TF-CBT)

Trauma-focused CBT is an integrated CBT program that is strongly recommended by guidelines. Shaw et al. [27, 28] conducted two experiments on TF-CBT interventions for preterm mothers. The study using group TF-CBT, which involved one or two 90-min sessions per week for 3–6 weeks [27], revealed that individual intervention was more effective than group intervention in reducing symptoms. Furthermore, the results immediately after treatment and through the 6-month follow-up showed that participants in the group intervention had significant improvement in trauma symptoms, as compared to those receiving individual TF-CBT in their prior study. In another study combining TF-CBT with techniques developed to help increase parenting confidence and change negative parental perceptions of preterm infants, participants who received the intervention scored remarkably lower on the (Davidson Trauma Scale) DTS, as compared to participants in the control group [28]. Similarly, Simon et al. [29] found that the mean trauma score of participants was marginally below the PTSD cut-off level before follow-up and significantly below the cut-off level at the end of the 6-month follow-up. Interestingly, no significant change in trauma symptoms was found from baseline to 6 weeks after intervention, but significant improvement in symptoms was found at 6 months of follow-up compared to baseline and 6 weeks of follow-up.

### Internet-based Cognitive Behavioral Therapy (iCBT)

Internet-based cognitive behavioral therapy consists of three phases. The first phase is self-confrontation, in which the traumatic loss and its circumstances are described, the second phase is cognitive reorganization, which aims to provide a new perspective on the loss, and the third phase is social sharing, which focuses on a symbolic farewell letter to the participants themselves, people involved in the loss, or their loved ones. Each writing session lasts for 45 min and is scheduled on a regular basis. In each session, the therapist provides feedback to individuals as well as instructions for the next writing task. Kersting et al. [26, 30] conducted studies on two iCBT interventions on people with miscarriage, termination of pregnancy due to fetal abnormalities, or stillbirth, which showed that the treatment group had significant improvement compared to the control group and that the improvement persisted through 3 months of follow-up. For participants receiving other interventions, 77.3% with a baseline diagnosis of PTSD improved and no longer met the diagnostic criteria for PTSD, while the percentage was only 31.7% for participants who did not receive the intervention.

## Eye Movement Desensitization Reprocessing (EMDR)

EMDR improves patients’ symptoms using a series of eye movements at different times in sequence. By linking the patients’ traumatic memories to bilateral alternating stimuli, EMDR can help change the cognitive processes of memory, thereby relieving trauma-related distress. Appelman-Dijkstra [31] reported a 34-year-old woman with a history of hyperparathyroidism and gestational hypercalcemia who was diagnosed with hyperemesis gravidarum at 2 weeks of gestation, HELLP syndrome at 38 weeks, and PTS symptoms after delivery, which improved after EMDR intervention. Similar conclusions were reached by Stramrood [32], who reported a patient diagnosed with pre-eclampsia at 1 week after her first delivery and postpartum hemorrhage after her second delivery; the patient’s PTS symptoms improved significantly after four EMDR sessions.

## Written exposure therapy

Expressive writing is based on inhibition theory, which links traumatic events to positive health outcomes. In this therapy, individuals are asked to write down their deepest thoughts and feelings from a painful or traumatic event they encountered. Generally, the writing session lasts for 15 min, is performed in a private space, and is scheduled for three consecutive days, with no feedback provided. Using a sample of preterm women, Horsch et al. [33], found a marked decrease in PTSD symptoms in the intervention group after 3 and 4 months of intervention, as compared to the control group, who only showed decreasing symptoms between 3 and 6 months after intervention. Taken together, evidence has shown that expressive writing is a simple, easily accessible, and cost-effective therapy that reduces maternal post-traumatic and depressive symptoms and improves the mental health of preterm infants after birth.

## Acceptance and Commitment Therapy (ACT)

ACT is an acceptation-based intervention usually consisting of seven modules and lasting for 7 days. The modules include short readings explaining concepts of ACT, often using metaphors, and one to two exercises such as driving your life bus and loving mercy meditation. Through a study on women with PPROM, Tunnell et al. [34] found that those who participated in ACT had no deterioration, as assessed using PCL-5 (PTSD Checklist for DSM-5). Despite the limitations of this study, ACT has been widely accepted regarding its feasibility and acceptability as a transaction-based ACT.

## Psychological grief counseling

Psychological grief counseling includes the understanding of stages of grief and the grief cycle, exposure to negative thoughts and practice of recovery writing, the thought-emotion cycle (involving cognitive reorganization and searching for meaning), and the practice of coping skills. It has shown a significant positive effect on reducing the severity of grief symptoms. Navidian et al. [35] found that for women with stillbirths, those who received psychological grief counseling had greater improvement in PTS symptoms than the control group.

## Early intervention programs

The early intervention program consists of three main aspects, i.e., parental support, parent–child relationship support, and infant development support. In a study on women with preterm birth, Borghini et al. [36] conducted an early intervention program, which was based on a treatment model involving the conversion from a family system theory into a transactional preventive intervention; the program included three phases and was scheduled at 33 weeks and 42 weeks after conception as well as 4 months after the theoretical term of 40 weeks after conception (i.e., corrected age). In this program, parents were encouraged to comment on their babies’ behavior and their own emotions and participate in a 10-min mother-infant interactive game; the whole process was recorded for the instructor to provide feedback. Each session lasted 40–60 min and was designed to improve the quality of maternal care and sensitivity to the infant by providing directions on observing the infant’s responses, needs, abilities, and weaknesses. The study found that the PTSD symptoms of mothers of preterm infants were significantly more severe than those of mothers of full-term infants, and the difference persisted through 12 months after the birth of their children.

## Family support program

The family support program focuses on emotions through real-time online counselling. In this program, the family function is evaluated after admission, and the family support plan is then formulated after communication with the patients and their families regarding the psychological characteristics of the pregnant women. Sun et al. [37] found that the family support program could improve PTSD symptoms in perinatal women, and another study of this group [38] indicated that the PTSD and hyperarousal symptoms of the participants who received family support intervention were significantly relieved, compared to those in the control group treated with routine care. In a study, Wang et al. [39] combined empathy nursing (focusing on active listening, understanding, respect, and acceptance) with a family support plan, which exhibited remarkable effects on pregnant women with fetal abnormalities. Sun et al. and Wu et al. [37, 40] conducted a family empowerment educational intervention for women with preeclampsia, which showed that the PCL-C (PTSD Checklist-Civilian Version) score of the participants was significantly reduced after intervention. Moreover, the severity of PTSD in patients with preeclampsia is closely related to family support. Dong et al. [41] found that the post-traumatic separation, depression and anxiety, and bereavement of patients were significantly reduced at 7 weeks after labor compared to 4 weeks after labor.

## Peer support

The peer support group is usually formed by pregnant women admitted to hospital for labor. Zhang et al. [42] found that peer support could effectively reduce PTSD symptoms in pregnant women with fetal abnormalities, and the symptoms of intrusion and hyperarousal were also improved after intervention, However, the participants’ avoidance failed to improve significantly.

## Mindfulness yoga

Yoga can help individuals maintain focused and calm and is suitable for women who refuse to take medications during pregnancy. For women who have experienced a stillbirth, yoga is an effective approach to reduce their mental and physical stress [43, 44]. Yoga can also improve sleep quality, reduce rumination and hyperarousal, and improve the ability to fall asleep and maintain sleep [45–47]. Using an online yoga intervention (60 min/week, 12 weeks) for women who had a stillbirth in the past 24 months, Huberty et al. found that PTS symptoms and associated sleep disturbances significantly improved [43].

## Music therapy

Music therapy mainly involves listening to music, singing, or playing musical instruments. Kobus et al. [48] found that the perceived distress of preterm birth (measured by Impact of Event Scale-Revised score) was significantly decreased in participants who received music therapy, as compared to the control group; music therapy could also improve hyperarousal symptoms effectively.

### Risk of bias

Data on the risk of bias for the included RCTs are shown in Figs. 2 and 3. All included studies were deemed to have a low risk of bias due to allocation concealment and low risk of bias in the selection of the reported outcome. The overall risk of bias was low for two of the 12 RCTs (studies 12, 20). Convenience sampling was used in three studies (studies 11, 13, 21), and six studies (studies 8, 11, 12, 14, 18, 21) had insufficient details about how the random sequence was generated. In the study by Zhang et al. (study 6), the trial design, by which participants were assigned to different groups based on their maternity unit rather than using a random numerical table, might have led to a higher risk of selection bias. A higher risk of follow-up bias was found in studies by Borghini and Kobus et al. In the study of Borghini et al. (study 15), the non-intervention group lacked follow-up data at 4 months, and in the study of Kobus et al. (study 18), up to 24% of participants dropped out during the course of the study. It should be noted that in the study of Kersting et al. (study 9), most of the participants were well-educated and did well in expressive writing; however, the result might be different for people with lower levels of education.

Information about the risk of bias in the three B-A studies (studies 2, 7, 17) is presented in Table 2. As we could not confirm that all subjects who met the entry requirements were enrolled, the sample size was insufficient to produce reliable results (studies 2, 17), and as the outcome measures were not evaluated before and after intervention, all the B-A studies were regarded as having a high risk of bias. The risk of bias for the observational cohort (study 5), cross-sectional (studies 1, 10) and case series (studies 3–4) studies is presented in Table 3 and Table 4.

**Table 2:** Risk of bias assessment for included before-after studies

		YingWu et al	Dong et al	Shaw et al
Questions	1. Was the study question or objective clearly stated?	Yes	Yes	Yes
	2. Were eligibility/selection criteria for the study population prespecified and clearly described?	Yes	Yes	Yes
	3. Were the participants in the study representative of those who would be eligible for the test/service/intervention in the general or clinical population of interest?	No	Yes	No
	4. Were all eligible participants that met the prespecified entry criteria enrolled?	Cannot determine	Cannot determine	Cannot determine
	5. Was the sample size sufficiently large to provide confidence in the findings?	No	Yes	No
	6. Was the test/service/intervention clearly described and delivered consistently across the study population?	Yes	Yes	Yes
	7. Were the outcome measures prespecified, clearly defined, valid, reliable, and assessed consistently across all study participants?	Yes	Yes	Yes
	8. Were the people assessing the outcomes blinded to the participants’ exposures/interventions?	Not applicable	Not applicable	Not applicable
	9. Was the loss to follow-up after baseline 20% or less? Were those lost to follow-up accounted for in the analysis?	Yes	Yes	No
	10. Did the statistical methods examine changes in outcome measures from before to after the intervention? Were statistical tests done that provided p values for the pre-to-post changes?	Yes	Yes	Yes
	11. Were outcome measures of interest taken multiple times before the intervention and multiple times after the intervention (i.e., did they use an interrupted time-series design?)	No	No	No
	12. If the intervention was conducted at a group level (e.g., a whole hospital, a community, etc.) did the statistical analysis take into account the use of individual-level data to determine effects at the group level?	Not applicable	Not applicable	Not applicable
	Quality rating	Poor	Poor	Poor

**Table 3:** Quality scoring for included observational cohort and cross-sectional studies

		Wang et al	Hubertyl et al	Kjeldgaard et al
Questions	1. Was the research question or objective in this paper clearly stated?	Yes	Yes	Yes
	2. Was the study population clearly specified and defined?	Yes	Yes	Yes
	3. Was the participation rate of eligible persons at least 50%?	Yes	Yes	Yes
	4. Were all the subjects selected or recruited from the same or similar populations (including the same time period)? Were inclusion and exclusion criteria for being in the study prespecified and applied uniformly to all participants?	Yes	Yes	Yes
	5. Was a sample size justification, power description, or variance and effect estimates provided?	Yes	Yes	Yes
	6. For the analyses in this paper, were the exposure(s) of interest measured prior to the outcome(s) being measured?	Yes	Yes	Yes
	7. Was the timeframe sufficient so that one could reasonably expect to see an association between exposure and outcome if it existed?	Yes	Yes	Yes
	8. For exposures that can vary in amount or level, did the study examine different levels of the exposure as related to the outcome (e.g., categories of exposure, or exposure measured as continuous variable)?	No	Yes	Yes
	9. Were the exposure measures (independent variables) clearly defined, valid, reliable, and implemented consistently across all study participants?	NR	NR	NR
	10. Was the exposure(s) assessed more than once over time?	No	No	Yes
	11. Were the outcome measures (dependent variables) clearly defined, valid, reliable, and implemented consistently across all study participants?	Yes	Yes	Yes
	12. Were the outcome assessors blinded to the exposure status of participants?	Not applicable	Not applicable	Not applicable
	13. Was loss to follow-up after baseline 20% or less?	Not applicable	Not applicable	Not applicable
	14. Were key potential confounding variables measured and adjusted statistically for their impact on the relationship between exposure(s) and outcome(s)?	No	No	Yes
	Quality rating	Poor	Poor	Poor

*NR* not reported

**Table 4:** Quality scoring for included case series studies

		Stramrood et al	POEL et al
Questions	1. Was the study question or objective clearly stated?	Yes	Yes
	2. Was the study population clearly and fully described, including a case definition?	Yes	Yes
	3. Were the cases consecutive?	Yes	Yes
	4. Were the subjects comparable?	Yes	Yes
	5. Was the intervention clearly described?	Yes	No
	6. Were the outcome measures clearly defined, valid, reliable, and implemented consistently across all study participants?	No	No
	7. Was the length of follow-up adequate?	Yes	Not applicable
	8. Were the statistical methods well-described?	Yes	No
	9. Were the results well-described?	No	No
	Quality rating	Fair	Poor

## Discussion

Pregnancy-related complications have been recognized as traumatic events that can result in PTSD, it has become a concern for clinicians.

### Hypertensive Disorders of Pregnancy (HDP)

HDP mainly manifests as hypertension, edema, proteinuria, etc. In severe cases, patients can also experience nausea, vomiting, headache, convulsion, coma, and other symptoms, which is an important cause of maternal and fetal death [49]. Wang et al. found that the positive rate of PTSD in women with HDP was 13.04% as assessed using PCL-C. The incidence of postpartum depression, anxiety, and PTSD in women with HDP is also found higher than that in women without HDP. Women with a history of HDP are more likely to develop PTSD compared to those with normal labor, and the severity of HDP may be correlated with the seriousness of the symptoms [50]. However, a shortcoming is the absence of psychological interventions specifically for PTSD patients afflicted with HDP.

### Pre-eclampsia (PE)

PE is a multisystem disorder of pregnancy characterized by concurrent hypertension (occurring after 20 weeks of gestation), proteinuria, and associated organ dysfunction [51]. Meanwhile, it is one of the major causes of maternal and perinatal mortality [52]. A large number of women who have experienced PE report their labor as a traumatic event [53]. Wu et al. [40] found that PTSD symptoms were significantly more severe at 1 day after childbirth than at admission among women with PE. It has been found that the prevalence of PTSD in women with PE ranges from 44 to 4% at 6 weeks to 5 years postpartum [54]. Moreover, compared to women with mild PE, those with severe PE are more likely to have PTSD, with a prevalence of 11% [55]. Stramrood et al. [32] reported that a 25-year-old woman with postpartum eclampsia who was diagnosed 1 week after her first delivery was found to have typical symptoms of PTSD, such as flashbacks of postpartum hospitalization, avoiding activities, places, and conversations related to childbirth. It was also found that PTSD symptoms were more severe in patients with PE than in those with HDP [50]. The most common symptom of PTSD was decrease in interest, which is present in approximately 56% of all cases [56].

### HELLP syndrome

HELLP syndrome is usually defined as a severe form of PE and is also known as “atypical pre-eclampsia”. It is characterized by hemolysis, microangiopathic hemolytic anemia, elevated liver enzymes, and low platelet levels. Many studies have found that the incidence of mental health problems has increased in patients with PE/HELLP. In a study by Poel et al. [57], among the Caucasian primiparas with PE, eclampsia, and/or hemolysis, and HELLP syndrome, 5 subjects were diagnosed with PTSD, 15 reported hyperarousal symptoms, 10 reported anxiety and feelings of tension, and 9 reported re-experience of their problems/symptoms. Patients diagnosed with PE or HELLP syndrome who developed PTSD or PTSS were treated with distinct psychological interventions, including family-involved empowerment education and EMDR therapy. Patients with PTSD showed varying degrees of improvement when treated with different interventions.

### Hyperemesis Gravidarum (HG)

HG refers to serious and persistent nausea and vomiting in early pregnancy, causing dehydration, ketosis, and even acidosis, which may require hospitalization. Kjeldgaard et al. [58] found that HG can also be a traumatic experience. Women with HG score had higher IES-R score at 8 weeks and 2 years postpartum than women with no, mild, or severe nausea. Women with HG are also at a higher risk of PTSD than those with premature childbirth. Unfortunately, studies on psychotherapy that aimed to effectively manage PTSD resulting from HG are still insufficient.

### Fetal abnormalities and miscarriage

Fetal abnormalities include congenital malformations (birth defects) and stillbirths, which often require hospitalization for induction of labor. The sudden occurrence of such events may bring unimaginable pain to the pregnant woman physically and mentally as well as potential psychological trauma through years after the events. Several studies have found the baseline IES-R score of pregnant women with fetal abnormalities is generally above 19, suggesting that this population needs increasing clinical attention [37, 38, 42]. Compared to women giving birth to a live infant, those with stillbirths were found to have a 12-fold increased risk for PTSD [59], indicating that stillbirths can be traumatic events. It has also been reported the strong symptoms of sadness, poor coping, hopelessness, worthlessness, isolation, and guilt may persist for up to 10 years after the stillbirth [33]. The study by Huberty et al. [60] has shed more light on women who experienced stillbirth; it showed that the majority of participants (*n* = 38) reported high levels of PTSD symptoms (IES-R ≥ 35). This result is also supported by the findings of Navidian et al. [35]. A study found that among patients who experienced late fetal death, the prevalence of PTSD ranged from 0.6% to 39% [61]. Furthermore, evidence suggests that miscarriage can increase the risk of PTSD among pregnant women [26, 30, 39]. A study found that 44.7% of the subjects [62] were at a greater risk of PTS after miscarriage. Women who experienced induced labor after an adverse pregnancy are found more likely to develop dissociative symptoms, with 20% of women in the study showing acute stress symptoms [41]. Studies using the IES-R scale to measure the risk of PTSD showed that women with fetal abnormalities and miscarriages scored significantly higher, generally above 30 (with critical values set at 22 to 40). Most therapies for fetal abnormalities and mortal abortions are based on CBT, and 44% (4/9) of the trials included in the present study are Internet-based. This might be related to participants’ reluctance to receive in-person treatment due to their concerns about confidentiality or other people’s opinions on their help-seeking [63]. Internet-based psychotherapy allows more privacy and flexibility as well as avoids high costs and geographical limitations. For instance, participants in iCBT sessions can use the material at any time based on their progress, which is deemed as an advantage over traditional face-to-face CBT [64]. One-third of the studies included in the present review used family support therapy, highlighting the significant link between post-partum family support and PTSD [65]. Due to its organized structure and focus on problem-solving, family support therapy can help individuals make better decisions and reduce negative perceptions of stressful events, thereby improving their symptoms of PTSD [66].

### Preterm, low birth-weight infants

Preterm birth can be a traumatic event for parents [67], and it may also impact mother-infant attachment and infant development [68]. Studies have found that parents can experience significant stress and even PTSD symptoms when their infants have to be separated from them and admitted to the NICU, as they worry that preterm birth will cause sequelae or even death to their infants [36]. Among the nine studies on preterm birth included in the present review, Borghini et al. [36] found that mothers of preterm infants (gestation age < 33 weeks) had significantly more severe PTSD symptoms than mothers of full-term infants. Preterm birth is often associated with low birth weight, both of which can bring traumatic effects and increased risk of PTSD to pregnant women [69]. Shaw et al. [27, 28] found that mothers of preterm infants (gestational age of 25–34 weeks; > 600 g) scored higher on scales of PTSD, and 90.3% of them were positive for acute stress reaction symptoms. Simon et al. [29] revealed that the mean trauma score of mothers of preterm infants (23–34 weeks of gestational age) at baseline was above the recommended clinical cut-off score for PTSD.

Studies on CBT have shown that this therapy could significantly reduce the severity of anxiety and PTSD in mothers of premature infants [70]. Furthermore, it can also reduce the symptoms of insomnia and depression as well as the risk of premature birth [71]. A study using group CBT showed a significant decrease in the severity of PTSD in participants after six months of intervention, with an average reduction of 22.26 points on the DTS scale. Studies have also found that therapies delivered in a time-limited, highly organized group style may bring persistent psychological benefits. Compared to individual TF-CBT, mothers participating in group CBT are more likely to feel more secure and supported and are more willing to share their experiences. Group CBT can also reduce the strain on mental health workers in the NICU setting. However, through the comparison of two studies, Shaw et al. suggested that both individual and group TF-CBT could significantly improve traumatic symptoms, but individual treatment showed a better effect.

For women who had experienced early childbirth, music therapy and expressive writing have been shown helpful. According to Kobus et al., music therapy showed a significantly better effect than conventional stress-reduction measures in the NICU for treating high awareness in preterm women; they also noticed that music therapy could be an additional stress-reducing approach in the NICU. The findings of Carr et al. revealed clinically significant improvement in the main symptoms of PTSD after music therapy, especially avoidance [72]. Another study found that music therapy performed by parents themselves could improve psychological outcomes [73]. Expressive writing is also helpful for mental health improvement, emotional ventilation, and rationalization of thoughts around abortion [44]. A study found that the subjects’ PTSD symptoms were significantly relieved through 3 to 4 months after they participated in expressive writing sessions. This is also supported by the study of Blasio et al., which found that subjects receiving expressive writing intervention for three months exhibited significantly decreased depression and posttraumatic symptoms [74].

## Strengths and limitations

To our knowledge, the present review is the first one to address the impact of pregnancy-related complications on maternal PTSD. Furthermore, the thorough search of databases has also allowed for comprehensive analysis of different interventions and different populations.

Overall, the quality of the involved studies was mixed, particularly in terms of study design and measurement of exposure and outcome data. As different studies involved participants in different stages of their perinatal period and participants with different PTSD-related diagnoses, the studied population of this review can be heterogeneous. Furthermore, as the effect of intervention was not assessed in some studies (studies 3, 10) and the sample size was small for some studies (studies 3, 4, 6, 11, 17–19), our results might not be generalizable to other populations. Moreover, PTSD symptoms were evaluated using self-reported questionnaires (studies 1–2, 5–13, 15–21) in most studies, which might have reduced the accuracy of our findings due to participants’ propensity to provide socially acceptable answers. The tools used to assess or diagnose PTSD also varied across studies, which might have affected the accuracy of our results. The lack of long-term follow-up may be another limitation. Among all the included studies, only 3 trials (studies 5,9,15) included a post-intervention assessment at 12 or 24 months. As the B-A studies (studies 2, 7, 17, 19) do not have a control group, they could not determine whether the improvement in symptoms was caused by the intervention or other factors such as the normal healing process [29]. Articles published in languages other than English and Chinese might have been overlooked as we restricted the scope of this study to articles published in English and Chinese. In general, more RCTs are still needed to investigate the association between maternal PTSD and pregnancy problems.

## Conclusion

In this scoping review, we evaluated studies on the effects of pregnancy-related complications on PTSD in women in the perinatal period and summarized the effective psychological treatment options. According to our analysis, a variety of pregnancy-related complications have been recognized as traumatic events that can cause short- or long-term consequences for women in the perinatal period. Therefore, more attention should be given to the mental health of this special population. More studies, especially high-quality RCTs, are still warranted to confirm the effects of different interventions on women with different pregnancy complications, as this may help maximize the effects, acceptability, and feasibility of the interventions.

### Supplementary Information

Supplementary Material 1.

## Data Availability

No datasets were generated or analysed during the current study.
