# Supplementary material for: Psychological interventions to pregnancy-related complications in patients with post-traumatic stress disorder: a scoping review
Source: BMC Psychiatry. 2024 Jun 27;24:478. doi: 10.1186/s12888-024-05926-6 (PMC11212442; doi:10.1186/s12888-024-05926-6)
Supplement: Supplementary file 1 — Supplementary Material 1. [file 12888_2024_5926_MOESM1_ESM.docx]

supplementary Table 1. Full search strategies for all databases and websites consulted during the selection process of included studies

| **PubMed (https://pubmed.ncbi.nlm.nih.gov/)** |
| --- |
| A: ("Stress Disorders, Post-Traumatic"[Mesh] OR PTSD[tiab] OR Post-Traumatic Stress Disorder[tiab] OR Post-Traumatic Stress Disorders[tiab] OR Post Traumatic Stress Disorders[tiab] OR Posttraumatic Stress Disorders[tiab] OR Posttraumatic Stress Disorder[tiab] OR Stress Disorder, Posttraumatic[tiab] OR Stress Disorders, Posttraumatic[tiab] OR Post Traumatic Stress Disorder[tiab] OR Stress Disorder, Post Traumatic[tiab] OR Stress Disorder, Post-Traumatic[tiab]) |
| B: ("Pregnant Women"[Mesh] OR Woman, Pregnant[tiab] OR Women, Pregnant[tiab] OR Pregnancy[tiab] OR Pregnancies[tiab] OR Gestation[tiab] OR Maternity[tiab] OR Perinatal[tiab] OR Prenatal[tiab] OR Postpartum[tiab]) |
| C: ("psychotherapy"[MeSH Terms] OR "behavior therapy"[MeSH Terms] OR "psychotherap*"[MeSH Terms] OR (("cognitive"[Title/Abstract] OR "behavio*"[Title/Abstract] OR "integrative"[Title/Abstract] OR "interpersonal"[Title/Abstract] OR "psychodynamic"[Title/Abstract] OR "acceptance"[Title/Abstract] OR "commitment"[Title/Abstract]) AND "therap*"[Title/Abstract])) |
| Final search strategy: A and B and C |
| **Embase (https://www.embase.com/)** |
| #1: 'posttraumatic stress disorder'/exp OR 'posttraumatic stress disorder' OR 'posttraumatic stress':ti,ab,kw OR 'posttraumatic syndrome':ti,ab,kw OR 'psychosis, posttraumatic':ti,ab,kw OR 'ptsd':ti,ab,kw OR (ptsd:ti,ab,kw AND 'posttraumatic stress disorder':ti,ab,kw) OR 'stress disorders, post-traumatic':ti,ab,kw OR (('stress disorders, traumatic':ti,ab,kw OR 'stress, posttraumatic':ti,ab,kw OR trauma:ti,ab,kw) AND 'stressor related disorders':ti,ab,kw) OR 'traumatic stress':ti,ab,kw OR 'traumatic stress disorder':ti,ab,kw OR 'traumatic stress disorders':ti,ab,kw OR 'posttraumatic stress disorder':ti,ab,kw |
| #2: 'pregnant woman'/exp OR 'pregnant woman' OR 'pregnant woman':ti,ab,kw OR 'child bearing':ti,ab,kw OR 'childbearing':ti,ab,kw OR 'gestation':ti,ab,kw OR 'gravidity':ti,ab,kw OR 'intrauterine pregnancy':ti,ab,kw OR 'pregnancy maintenance':ti,ab,kw OR 'pregnancy trimesters':ti,ab,kw OR 'pregnancy':ti,ab,kw OR 'gestational':ti,ab,kw OR 'perinatal':ti,ab,kw OR 'prenatal':ti,ab,kw |
| #3: 'psychotherapy'/exp OR psychotherapy OR 'behavior therapy':ti,ab,kw OR 'psychotherap*':ti,ab,kw OR (('cognitive':ti,ab,kw OR 'behavio*':ti,ab,kw OR 'integrative':ti,ab,kw OR 'interpersonal':ti,ab,kw OR 'psychodynamic':ti,ab,kw OR 'acceptance':ti,ab,kw OR 'commitment':ti,ab,kw) AND 'therap*':ti,ab,kw) |
| Final search strategy: #1 and #2 and #3 |
| **Cochrane (https://www.cochranelibrary.com/library)** |
| #1: MeSH descriptor: [Stress Disorders, Post-Traumatic] explode all trees |
| #2: MeSH descriptor: [Pregnant Women] explode all trees |
| #3: MeSH descriptor: [Psychotherapy] explode all trees |
| #4: (Acute Post-Traumatic Stress Disorder OR Acute Post Traumatic Stress Disorder OR Chronic Post-Traumatic Stress Disorder OR Chronic Post Traumatic Stress Disorder OR Posttraumatic Stress Disorder OR Post Traumatic Stress Disorders OR Stress Disorder, Posttraumatic OR Stress Disorders, Posttraumatic OR Stress Disorder, Post Traumatic OR Neuroses, Posttraumatic OR PTSD OR Neuroses, Post-Traumatic OR Post Traumatic Stress Disorder OR Post-Traumatic Stress Disorder OR Posttraumatic Stress Disorders OR Neuroses, Post Traumatic OR Post-Traumatic Neuroses OR Stress Disorder, Post-Traumatic OR Post-Traumatic Stress Disorders OR Posttraumatic Neuroses OR Delayed Onset Post Traumatic Stress Disorder OR Delayed Onset Post-Traumatic Stress Disorder):ti,ab,kw |
| #5: (Woman, Pregnant OR Women, Pregnant OR Pregnant Woman OR Pregnancy OR Pregnancies OR Gestation OR Maternity OR Perinatal OR Prenatal OR Postpartum):ti,ab,kw |
| #6: ("psychotherapies"):ti,ab,kw |
| #7: MeSH descriptor: [Behavior Therapy] explode all trees |
| #8: (Treatment, Behavior OR Conditioning Therapy OR Behavior Change Techniques OR Behavior Treatment OR Behavior Change Technique OR Therapy, Conditioning OR Behavior Therapies OR Conditioning Therapies OR Modification, Behavior OR Therapy, Behavior OR Technique, Behavior Change OR Behavior Modifications OR Behavior Modification):ti,ab,kw |
| #9: MeSH descriptor: [Psychotherapy, Psychodynamic] explode all trees |
| #10: (cognitive OR behavio* OR integrative OR interpersonal OR psychodynamic OR acceptance OR commitment):ti,ab,kw |
| #11: (therap*):ti,ab,kw |
| #12: #1 OR #4 |
| #13: #2 OR #5 |
| #14: (#3 OR #6) OR (#7 OR #8) OR #9 OR (#10 AND #11) |
| Final search strategy: #15: #12 AND #13 AND #14 |
| **ISI Web of Science (https://www.webofscience.com/)** |
| A: (TS=(PTSD) OR TI=(Acute Post-Traumatic Stress Disorder OR Acute Post Traumatic Stress Disorder OR Chronic Post-Traumatic Stress Disorder OR Chronic Post Traumatic Stress Disorder OR Posttraumatic Stress Disorder OR Post Traumatic Stress Disorders OR Stress Disorder, Posttraumatic OR Stress Disorders, Posttraumatic OR Stress Disorder, Post Traumatic OR Neuroses, Posttraumatic OR PTSD OR Neuroses, Post-Traumatic OR Post Traumatic Stress Disorder OR Post-Traumatic Stress Disorder OR Posttraumatic Stress Disorders OR Neuroses, Post Traumatic OR Post-Traumatic Neuroses OR Stress Disorder, Post-Traumatic OR Post-Traumatic Stress Disorders OR Posttraumatic Neuroses OR Delayed Onset Post Traumatic Stress Disorder OR Delayed Onset Post-Traumatic Stress Disorder) OR AB=(Acute Post-Traumatic Stress Disorder OR Acute Post Traumatic Stress Disorder OR Chronic Post-Traumatic Stress Disorder OR Chronic Post Traumatic Stress Disorder OR Posttraumatic Stress Disorder OR Post Traumatic Stress Disorders OR Stress Disorder, Posttraumatic OR Stress Disorders, Posttraumatic OR Stress Disorder, Post Traumatic OR Neuroses, Posttraumatic OR PTSD OR Neuroses, Post-Traumatic OR Post Traumatic Stress Disorder OR Post-Traumatic Stress Disorder OR Posttraumatic Stress Disorders OR Neuroses, Post Traumatic OR Post-Traumatic Neuroses OR Stress Disorder, Post-Traumatic OR Post-Traumatic Stress Disorders OR Posttraumatic Neuroses OR Delayed Onset Post Traumatic Stress Disorder OR Delayed Onset Post-Traumatic Stress Disorder)) |
| B: (TS=(pregnant woman) OR TI=(Woman, Pregnant OR Women, Pregnant OR Pregnant Woman OR Pregnancy OR Pregnancies OR Gestation OR Maternity OR Perinatal OR Prenatal OR Postpartum) OR AB=(Woman, Pregnant OR Women, Pregnant OR Pregnant Woman OR Pregnancy OR Pregnancies OR Gestation OR Maternity OR Perinatal OR Prenatal OR Postpartum)) |
| C: (TS=(psychotherapy) OR TS=(behavior therapy) OR TS=(psychotherap*) OR TI=((cognitive OR behavio* OR integrative OR interpersonal OR psychodynamic OR acceptance OR commitment) AND therap*) OR AB=((cognitive OR behavio* OR integrative OR interpersonal OR psychodynamic OR acceptance OR commitment) AND therap*)) |
| Final search strategy: A and B and C |
| **CNKI (https://kns.cnki.net/)** |
| Search: （(SU %= 'Post-traumatic stress disorder' OR TKA= 'Post-traumatic stress' OR TKA= ptsd') AND (SU%= 'psychotherapy' OR TKA =' psychotherapy 'OR TKA='psychological intervention' OR SU%='supplementary therapy' OR TKA='supplements' OR SU %='exercise therapy’ OR TKA='athletic therapy' OR TKA=‘Cognitive therapy’ OR TKA=‘behavioral therapy’ or TKA=‘Exposure therapy‘ OR TKA= ‘Eye Movement’） AND （SU%= Pregnancy OR TKA= Maternity OR TKA= Pregnant OR TKA= Maternal OR TKA=Generation OR TKA= Prenatal OR TKA=post-partum OR TKA = childbirth） |
| **WANFANG DATA (https://s.wanfangdata.com.cn/advanced-search/paper)** |
| Search: （subject:（Post-traumatic stress disorder） OR title or keywords:（Post-traumatic stress disorder OR Post-traumatic stress OR PTSD） OR abstract: （Post-traumatic stress disorder OR Post-traumatic stress OR PTSD）） AND（subject:（pregnant woman） OR title or keywords:（Pregnancy OR maternity OR pregnant women OR mother OR pregnant OR prenatal OR post-partum OR childbirth) OR abstract:（（Pregnancy OR maternity OR pregnant women OR mother OR pregnant OR prenatal OR post-partum OR childbirth)）AND （subject:（Psychotherapy OR Psychological interventions OR Supplementary therapy OR Exercise therapy ） OR title or keywords:（Supplementary therapy OR Exercise therapy OR mindfulness OR Cognitive therapy OR Behavioral therapy OR Exposure therapy OR Eye Movement）OR abstract:（Supplementary therapy OR Exercise therapy OR mindfulness OR Cognitive therapy OR Behavioral therapy OR Exposure therapy OR Eye Movement）） |
